# Clinical outcomes of concurrent repair of occult contralateral inguinal hernias during TAPP: a retrospective cohort study

**DOI:** 10.3389/fsurg.2026.1963908

**Published:** 2026-09-29

**Authors:** Hanxia Hao, Lijia Liu, Yao Wang, Simeng Chen, Yuan Li

**Affiliations:** 1 Department of Head & Neck Surgery, The Affiliated Cancer Hospital of Nanjing Medical University & Jiangsu Cancer Hospital & Jiangsu Institute of Cancer Research, Nanjing, China; 2 Department of General Surgery, The First Affiliated Hospital with Nanjing Medical University, Nanjing, China

**Keywords:** inguinal hernia, myopectineal orifice, occult hernia, surgical outcomes, transabdominal preperitoneal (TAPP) repair

## Abstract

**Background:**

To evaluate the clinical efficacy and surgical safety of intraoperative exploration and immediate repair of occult inguinal hernia (OIH) during transabdominal preperitoneal (TAPP) repair.

**Methods:**

A retrospective analysis was conducted on the clinical data of 238 adult patients who underwent TAPP at Jiangsu Provincial People’s Hospital (The First Affiliated Hospital with Nanjing Medical University) from May 2023 to May 2024. Based on preoperative diagnosis and intraoperative exploration findings, patients were divided into three groups: the unilateral group (without OIH, *n* = 173), the OIH group (intraoperatively explored and repaired, *n* = 32), and the preoperative bilateral group (*n* = 33). Operative time, total hospitalization costs, and the incidence of postoperative complications (including pain, seroma, urinary retention, and recurrence) were compared among the three groups.

**Results:**

Among patients preoperatively diagnosed with unilateral hernia, the intraoperative exploration rate of OIH was 15.61% (32/205). Indirect inguinal hernia was the predominant type of OIH (71.88%). The operative time in the OIH group (112.03 ± 37.61 min) was significantly longer than that in the unilateral group (*P* < 0.001), but showed no statistically significant difference compared with the preoperative bilateral group (*P* = 0.258). Total hospitalization costs in the OIH group (17119.57 ± 1958.58 CNY) were significantly higher than in the unilateral group (*P* < 0.001), but slightly lower than in the preoperative bilateral group (*P* = 0.010). Postoperative complication analysis revealed that the incidence of acute pain in the OIH group (31.25%) was significantly higher than in the unilateral group (*P* = 0.008). However, no statistically significant difference was observed when compared with the preoperative bilateral group (*P* = 0.221). No statistically significant differences were observed among the three groups in the incidence of other complications, such as seroma, urinary retention, and hernia recurrence.

**Conclusions:**

Routine intraoperative exploration during TAPP effectively identifies occult inguinal hernias. Concurrent laparoscopic repair of an incidentally detected OIH appears technically feasible and exhibits a favorable safety profile, without an obvious increase in major short-term postoperative complications compared with standard unilateral or bilateral repairs. These findings support concurrent repair as a feasible and safe intraoperative choice when an OIH was encountered.

## Introduction

Inguinal hernia was one of the most common conditions in general surgery, with surgical intervention remaining the definitive treatment. With advances in microanatomy and the widespread adoption of minimally invasive techniques, transabdominal preperitoneal (TAPP) repair has become a standard procedure globally due to its advantages of mild postoperative pain, rapid recovery, and low recurrence rates (1, 2).

Compared with traditional open techniques, the primary anatomical advantage of TAPP lies in its laparoscopic view, which enables simultaneous and clear visualization of the bilateral myopectineal orifices (MPO) without additional incisions (1). This capability facilitates the detection of occult inguinal hernia (OIH)—defined as a hernia absent on preoperative physical and radiological examinations but confirmed during intraoperative exploration.

Existing studies (1, 3) indicate that the incidence of contralateral OIH in patients with clinically unilateral inguinal hernia ranges from 13% to 22%. However, whether intraoperatively detected OIH warrants routine prophylactic repair remains controversial. While European Hernia Society (EHS) guidelines (4) previously suggested a “watchful waiting” strategy for asymptomatic occult hernias, accumulating clinical evidence (5, 6) demonstrates a high risk of progression to overt hernias within a few years, exposing patients to the physical trauma and financial burden of a second operation. Other studies (7, 8) also note that immediate intraoperative repair effectively reduces the incidence of metachronous contralateral hernia without significantly increasing surgical risks or complications.

Nevertheless, large-scale retrospective data regarding the long-term clinical efficacy, surgical safety, and health economics of repairing OIH discovered during TAPP remain limited. This study retrospectively analyzed clinical data from patients undergoing TAPP who were found to have and subsequently underwent repair of OIH at our institution. Therefore, the primary objective of this retrospective cohort study was to evaluate the technical feasibility, surgical safety, postoperative clinical outcomes, and hospitalization expenditure of concurrent repair of incidentally discovered OIH during TAPP, comparing these metrics with planned unilateral and bilateral TAPP repairs. This assessment aims to provide pragmatic clinical evidence to inform standardized intraoperative decision-making.

## Methods

### Study population

We retrospectively collected data from adult patients who underwent elective TAPP at Jiangsu Province Hospital (The First Affiliated Hospital with Nanjing Medical University) between May 1, 2023, and May 1, 2024.

**Inclusion criteria:** Preoperative diagnosis of inguinal hernia; Age 18–80 years; No prior history of inguinal hernia surgery; Written informed consent to participate in the study and consent to surgical repair if an occult hernia was discovered intraoperatively.

**Exclusion criteria:** Intraoperative conversion to open surgery or an alternative procedure; Preoperative incarcerated or strangulated hernia; Severe comorbidities precluding study participation; Patients deemed unsuitable for participation by investigators for other clinical reasons.

Patients were categorized into three groups:

Unilateral group (no OIH detected, underwent unilateral repair; *n* = 173);

OIH group (intraoperatively diagnosed with OIH and underwent bilateral repair; *n* = 32);

Preoperative bilateral group (preoperatively diagnosed with bilateral inguinal hernia and underwent bilateral repair; *n* = 33).

A total of 238 patients were included. All patients completed a minimum follow-up period of one year post-discharge, conducted through a combination of active outpatient clinical visits and telephone or online follow-ups administered by the investigators.

Demographic baseline characteristics, operative variables, and postoperative outcomes were retrieved from the Electronic Medical Record system of Jiangsu Province Hospital. The study protocol was approved by the Institutional Ethics Committee of Jiangsu Province Hospital (Approval No.: 2026-SR-233).

### Definition of occult inguinal hernia

An occult inguinal hernia (OIH) was defined as a hernia absent on preoperative clinical examination and routine preoperative imaging, but identified and confirmed during TAPP intraoperative exploration.

Preoperative Standardized Evaluation:

All patients underwent a standardized preoperative clinical evaluation performed independently by two experienced hernia specialists, consisting of physical examination in both standing and supine positions during resting and positive Valsalva maneuvers. Standardized high-frequency ultrasonography of the bilateral groin was routinely performed for all enrolled patients as the primary imaging modality. Contrast-enhanced or non-contrast computed tomography (CT) of the abdomen and pelvis was additionally utilized in selective cases with ambiguous clinical/ultrasound findings or complex abdominal surgical history. All preoperative images were formally interpreted by subspecialized radiologists following a standardized institutional protocol to confirm the absence of detectable hernia sacs or bowel/omental herniation prior to surgery.

Intraoperative Diagnostic Criteria:

During laparoscopic TAPP exploration, OIH was diagnosed under pneumoperitoneum visualization based on objective, standardized anatomical criteria consistently applied by the surgical team:

(1) a distinct peritoneal pouch, funnel-shaped depression, or anatomical defect at the internal deep ring or Hesselbach’s triangle with a measured defect diameter ≥0.5 cm;

or (2) prominent retroperitoneal fat herniation (cord lipoma) extending into the inguinal canal through the deep ring, accompanied by secondary enlargement or weakening of the myopectineal orifice fascia (9–11).

### TAPP procedure

All TAPP procedures were performed using a standardized three-port technique. A camera port was placed at the inferior umbilical margin, and two working ports were placed at the lateral border of the rectus abdominis muscle (one at the umbilical level on the affected side and one below the umbilical level on the contralateral side). Routine bilateral exploration of the inguinal region was conducted. If an OIH was confirmed, concurrent bilateral tension-free repair was performed based on prior informed consent. A lightweight 3DMax polypropylene mesh (10.3 cm × 15.7 cm; BARD, USA) was placed in the preperitoneal space to ensure full coverage of the MPO. After verifying proper mesh positioning, the peritoneum was sutured closed. All surgeries were performed by the same surgical team following this standardized protocol.

### Clinical outcome measures

Evaluated parameters included preoperative baseline variables (sex, age, body mass index [BMI], American Society of Anesthesiologists [ASA] physical status classification, prior lower abdominal surgery, diabetes mellitus, history of corticosteroid therapy, and use of anticoagulant or antiplatelet medications), intraoperative parameters (operative time, estimated blood loss), postoperative stay, total hospital expenditure, and postoperative complications [hernia recurrence, seroma, hematoma, surgical site infection (SSI), urinary retention, and postoperative pain].

Postoperative pain was quantitatively assessed using the Visual Analog Scale (VAS, 0-10). Clinically significant acute pain was defined as a VAS score≥4 that required supplemental systemic analgesic administration (e.g., nonsteroidal anti-inflammatory drugs, NSAIDs). Pain scores and analgesic requirements were systematically recorded at 24 h postoperatively and at discharge. Follow-up evaluations at 1, 3, and 12 months specifically assessed for chronic post-herniorrhaphy pain (CPIP), defined as persistent groin discomfort lasting＞3 months postoperatively.

### Follow-up protocol and outcome assessment

All enrolled patients were followed up according to a structured institutional protocol at 1, 3, 6, and 12 months postoperatively, and annually thereafter. The median follow-up duration for the entire cohort was 18 months (range: 12–36 months).

Follow-up was primarily conducted via scheduled in-person outpatient clinic visits, during which two independent hernia surgeons performed detailed physical examinations in both standing and supine positions with Valsalva maneuvers. Standardized telephone follow-ups using a structured questionnaire were utilized for patients unable to attend clinic visits due to distance or scheduling conflicts. If a patient reported groin discomfort, a palpable bulge, or pain during telephone contact, an immediate in-person clinical evaluation with high-frequency ultrasonography was mandated.

Hernia recurrence was defined as a palpable bulge or impulse in the operated groin region during physical examination, strictly confirmed by high-frequency ultrasonography or CT imaging showing peritoneal sac protrusion or mesh displacement.

Out of the initial 245 patients screened, 7 patients (2.86%) were lost to follow-up (4 due to unreachable contact details and 3 due to relocation), leaving 238 patients who successfully completed the minimum 12-month follow-up evaluation and were included in the final analysis.

### Statistical analysis

Statistical analysis was performed using SPSS software (version 31.0.0; IBM Corp., Armonk, NY, USA). Continuous variables were first evaluated for normality using the Shapiro–Wilk test. Continuous data conforming to a normal distribution were expressed as mean ± standard deviation (x ± s) and were compared among the three groups using one-way Analysis of Variance (ANOVA). When global ANOVA indicated statistical significance, Bonferroni *post-hoc* tests were applied for pairwise comparisons. Non-normally distributed continuous variables were presented as median (interquartile range) and were analyzed using the Kruskal–Wallis H test, followed by Dunn–Bonferroni *post-hoc* tests for pairwise evaluations. Categorical variables were reported as frequencies and percentages (%) and were evaluated using the overall Chi-square test or Fisher’s exact test across all three groups; subsequent pairwise comparisons were adjusted using Bonferroni correction (with an adjusted significance threshold of *α* = 0.05/3 = 0.0167). A two-tailed *P* < 0.05 (or adjusted *P* value for *post-hoc* tests) was considered statistically significant.

## Results

A total of 238 patients were enrolled: 173 (72.69%) in the unilateral group, 32 (13.45%) in the OIH group, and 33 (13.87%) in the preoperative bilateral group. Among patients initially diagnosed with unilateral inguinal hernia preoperatively, the intraoperative detection rate of OIH was 15.61%.

Comparing the unilateral group with the OIH and preoperative bilateral groups revealed no statistically significant differences in baseline characteristics, including sex, age, BMI, ASA grade, prior lower abdominal surgery, diabetes, corticosteroid use, and anticoagulant/antiplatelet therapy (Table 1).

**Table 1:** General characters of patients in each group.

Demographic and clinical characteristics	Unilateral group (*n* = 173)	OIH group (*n* = 32)	Preoperative bilateral group (*n* = 33)	*P*_1_Value (Unilateral/OIH)	*P*_2_Value (OIH/ Bilateral)	Global *P* Value
Sex (n/%)						
Male	152 (87.86%)	31 (96.88%)	29 (87.88%)	0.211	0.355	0.318
Female	21 (12.14%)	1 (3.12%)	4 (12.12%)			
Age (years)	52.39 ± 16.43	52.22 ± 18.63	59.79 ± 10.77	0.957	0.051	0.051
BMI (kg/m^2^)	23.67 ± 2.62	23.94 ± 3.00	24.17 ± 3.11	0.601	0.757	0.585
ASA Physical Status Classification (n/%)						
ASA I	38 (21.97%)	4 (12.50%)	4 (12.12%)	0.327	1.000	0.432
ASA II	115 (66.47%)	23 (71.88%)	27 (81.82%)	0.550	0.341	
ASA III	20 (11.56%)	5 (15.62%)	2 (6.06%)	0.725	0.258	
Prior lower abdominal surgery (n/%)	5 (2.89%)	2 (6.25%)	0	0.300	0.238	0.331
Diabetes mellitus (n/%)	10 (5.78%)	2 (6.25%)	1 (3.03%)	1.000	0.613	0.800
History of corticosteroid therapy (n/%)	1 (0.58%)	0	0	1.000	/	0.830
Use of anticoagulant or antiplatelet medications (n/%)	10 (5.78%)	4 (12.50%)	1 (3.03%)	0.316	0.197	0.254

In the OIH group (Table 2), 17 patients (53.13%) had left-sided OIH, with a mean defect diameter of 1.56 ± 0.70 cm. Anatomic types included 23 indirect hernias (71.88%), 7 direct hernias (21.87%), and 2 combined (pantaloon) hernias (6.25%).

**Table 2:** Clinical characteristics of OIH.

OIH (n)	32
Side (n/%)	
Left	17 (53.13%)
Right	15 (46.87%)
Defect diameter (cm)	1.56 ± 0.70
Types of hernias (n/%)	
Indirect inguinal hernias	23 (71.88%)
Direct inguinal hernias	7 (21.87%)
Combined (pantaloon) hernias	2 (6.25%)

**Table 3:** Intraoperative and postoperative data of patients in each group.

Perioperative and economic outcomes	Unilateral group (*n* = 173)	OIH group (*n* = 32)	Preoperative bilateral group (*n* = 33)	*P*_1_Value (Unilateral/OIH)	*P*_2_Value (OIH/ Bilateral)	Global *P* Value
Operative time (min)	71.39 ± 22.44	112.03 ± 37.61	121.64 ± 29.93	**<0**.**001**	0.258	**<0**.**001**
Estimated blood loss (ml)	4.71 ± 10.20	10.31 ± 16.75	13.33 ± 37.56	0.076	0.678	**0**.**016**
Postoperative stay (days)	1.46 ± 0.84	1.59 ± 0.76	1.88 ± 1.05	0.410	0.216	0.331
Total hospital expenditure (CNY)	13959.21 ± 1654.52	17119.57 ± 1958.58	18233.32 ± 1305.08	**<0**.**001**	**0**.**010**	**<0**.**001**

Bold values indicate statistical significance (*P* < 0.05).

Operative time was significantly longer in the OIH group than in the unilateral group (112.03 ± 37.61 min vs. 71.39 ± 22.44 min, *P* < 0.001), but did not differ significantly from the preoperative bilateral group (112.03 ± 37.61 min vs. 121.64 ± 29.93 min, *P* = 0.258) (Table 3). Total hospital expenditure in the OIH group (17,119.57 ± 1,958.58 CNY) was significantly higher than in the unilateral group (13,959.21 ± 1,654.52 CNY, *P* < 0.001), but slightly lower than in the preoperative bilateral group (18,233.32 ± 1,305.08 CNY, *P* = 0.010).

In the OIH group (Table 4), 2 patients developed seroma, 13 experienced urinary retention, and 10 reported noticeable postoperative pain. No cases of hernia recurrence, hematoma, or SSI occurred. The incidence of postoperative pain was significantly higher in the OIH group than in the unilateral group (31.25% vs. 12.72%, *P* = 0.008), but comparable to the preoperative bilateral group (31.25% vs. 18.18%, *P* = 0.221).

In our cohort, the incidence of acute postoperative pain was significantly higher in the OIH group than in the unilateral group (31.25% vs. 12.72%, *P* = 0.008), though comparable to the preoperative bilateral group (18.18%, *P* = 0.221). Incidence rates of other complications, including seroma, urinary retention, recurrence, hematoma, and SSI, showed no significant differences among the three groups.

## Discussion

Whether intraoperatively identified OIH warrants routine concurrent repair remains a topic of clinical discussion (3, 5). In our study, all patients with identified OIH underwent immediate repair. Although our single-arm repair design cannot directly prove superiority over watchful waiting, our findings demonstrate that concurrent repair was technically streamlined, achieving an operative duration (112.03 ± 37.61 min) comparable to the preoperative bilateral group (*P* = 0.258). Furthermore, concurrent OIH repair achieved a satisfactory cost profile compared with planned bilateral repair, avoiding the potential cumulative financial burden of two separate surgical admissions.

Accurate diagnosis of inguinal hernia was essential for formulating an appropriate surgical strategy. However, given the limited sensitivity of physical examination and conventional imaging for subtle hernia sacs or early-stage defects, OIH frequently leads to metachronous contralateral hernia development postoperatively, which was often misattributed to “recurrence” (12).

In this study, intraoperative TAPP exploration identified OIH in 15.61% of patients initially diagnosed with unilateral hernia, aligning closely with the 13%–22% rate reported in the HerniaSurge international guidelines (1). Anatomically, OIH in our study comprised predominantly indirect hernias (71.88%) with a small mean defect diameter (1.56 ± 0.70 cm). Such subtle defects were rarely detectable during physical examination in a non-pressurized state. The primary advantage of

TAPP bypasses the abdominal wall barrier to offer clear and direct laparoscopic visualization of the bilateral myopectineal orifices. Through pneumoperitoneum-induced markers such as peritoneal depression or deep ring funneling, TAPP effectively facilitates the identification of occult defects that were undetectable during physical or radiological examinations (13).

In terms of direct medical expenditure within this cohort, total hospitalization costs in the OIH group (17,119.57 ± 1,958.58 CNY) were significantly higher than those in the unilateral group (13,959.21 ± 1,654.52 CNY, *P* < 0.001), but remained slightly lower than those in the preoperative bilateral group (18,233.32 ± 1,305.08 CNY, *P* = 0.010). Although a formal health-economic cost-effectiveness analysis was not performed, and long-term indirect costs such as future potential readmissions or productivity loss were not prospectively measured. Our data reflect that concurrent repair increases initial single-admission costs relative to unilateral repair while avoiding the secondary facility and anesthesia fees associated with a separate future operation.

The incidence of acute postoperative pain (VAS≥4) was significantly higher in the OIH group than in the Unilateral group (31.25% vs. 12.72%, *P* = 0.008), but comparable to the Preoperative Bilateral group (18.18%, *P* = 0.221). This increased rate of early pain in the OIH group was attributable to the anatomical and surgical requirements of bilateral TAPP repair, which involve bilateral preperitoneal dissection, extensive peritoneal flap mobilization, and longer peritoneal closure suture lines inducing greater somatic and visceral peritoneal traction pain during the immediate postoperative window (24–48 h). Importantly, all reported pain events in Table 4 represent transient acute postoperative pain. All affected patients were successfully managed with oral NSAIDs (14) and experienced complete symptom resolution within 1 week post-discharge (15). During the 1-year follow-up period, zero cases of chronic post-herniorrhaphy pain (> 3 months) were identified in any group, supporting the long-term neural safety of using anatomically contoured 3DMax lightweight meshes without mechanical tack fixation.

**Table 4:** Postoperative complications data of patients in each group.

Postoperative complications	Unilateral group (*n* = 173)	OIH group (*n* = 32)	Preoperative bilateral group (*n* = 33)	*P*_1_Value (Unilateral/OIH)	*P*_2_Value (OIH/ Bilateral)	Global *P* Value
Hernia recurrence (n/%)	0	0	0	/	/	/
Seroma (n/%)	11 (6.36%)	2 (6.25%)	1 (3.03%)	1.000	0.613	0.757
Hematoma (n/%)	1 (0.58%)	0	0	1.000	/	0.830
Surgical site infection (n/%)	0	0	0	/	/	/
Urinary retention (n/%)	46 (26.59%)	13 (40.63%)	14 (42.42%)	0.107	0.883	0.083
Acute postoperative pain (n/%)	22 (12.72%)	10 (31.25%)	6 (18.18%)	**0**.**008**	0.221	**0**.**029**

Bold values indicate statistical significance (*P* < 0.05).

Complication rates for seroma, urinary retention, and recurrence were stable in the OIH group. The absence of recurrence highlights that adequate mesh coverage of the entire MPO yields durable repair, even over subtle anatomical weaknesses unrecognized preoperatively.

Based on current literature (15–17) and our findings, OIH represents a true anatomical defect rather than a physiological variation, with negligible potential for self-resolution. Because laparoscopic contralateral exploration requires no additional incisions, concurrent repair serves as a practical intraoperative strategy that eliminates the risk of future metachronous hernia development.

Contextualizing our findings within the most recent literature, our results strongly complement a 2026 systematic review and single-arm meta-analysis (12) (comprising 4,485 patients across 5 studies), which established that contralateral OIH was identified in nearly one in five patients (19.5%) undergoing minimally invasive repair, strictly aligning with our observed 15.61% detection rate and supporting routine intraoperative exploration during TAPP. Regarding repair strategy and safety, while observation risks secondary metachronous operations, concurrent OIH repair demonstrates a highly favorable safety profile: the 2026 meta-analysis (12) reported a overall complication rate of 4.7% (pooled 13%), negligible SSI (0.4%), and zero mortality or reoperations. This matches our cohort’s low complication profile, where increased acute pain (31.25%) and operative time represented a manageable transient burden rather than severe morbidity. Furthermore, our zero 1-year recurrence and zero chronic post-herniorrhaphy pain (CPIP) rates support the preliminary durability of concurrent repair, complementing the 2026 review’s low recurrence rate (2.2% at 30 months). While concurrent OIH repair slightly elevates initial single-admission costs and operative time, its safety and single-stage resolution of bilateral defects justify its role as a practical intraoperative strategy, though prospective randomized trials remain necessary to confirm long-term cost-effectiveness.

This study has several inherent limitations. First, because all patients with intraoperatively detected OIH underwent immediate repair based on prior consent, our cohort lacks an untreated observation control group to directly quantify the risk reduction of metachronous hernia. Second, the relatively modest sample size of the OIH group ($n = 32$) limits statistical power for detecting rare adverse events; thus, the absence of statistically significant differences should not be construed as definitive clinical equivalence. Finally, although all patients completed a minimum 12-month follow-up (median: 18 months), this timeframe remains relatively short for capturing late-onset recurrences. Future prospective, multi-center randomized controlled trials with multi-year follow-up are warranted.

## Conclusion

In conclusion, routine contralateral exploration during TAPP repair provides high diagnostic sensitivity for occult inguinal hernias. Immediate concurrent laparoscopic repair of OIH is technically straightforward and surgically safe, showing no significant increase in major operative complications or short-term recurrence compared with standard unilateral or bilateral repairs. Although concurrent repair incurs modest increases in operative time and single-admission expenses relative to unilateral repair, its safety and single-anesthesia approach support its clinical utility. When an occult inguinal hernia is identified during TAPP, concurrent repair is a practical and safe intraoperative strategy.

## Data Availability

The datasets presented in this article are not readily available because the dataset contains sensitive clinical information from electronic medical records. Access is restricted to protect patient privacy and comply with the regulations of the Institutional Ethics Committee of Jiangsu Province Hospital. De-identified data may be made available by the corresponding author upon reasonable request and ethical approval. Requests to access the datasets should be directed to drliyuan@njmu.edu.cn.
